# Spinnability and Characteristics of Polyvinylidene Fluoride (PVDF)-based Bicomponent Fibers with a Carbon Nanotube (CNT) Modified Polypropylene Core for Piezoelectric Applications

**DOI:** 10.3390/ma6072642

**Published:** 2013-07-03

**Authors:** Benjamin Glauß, Wilhelm Steinmann, Stephan Walter, Markus Beckers, Gunnar Seide, Thomas Gries, Georg Roth

**Affiliations:** 1 Institut für Textiltechnik der RWTH Aachen (ITA), Aachen 52056, Germany; E-Mails: wilhelm.steinmann@ita.rwth-aachen.de (W.S.); stephan.walter@ita.rwth-aachen.de (S.W.); markus.beckers@ita.rwth-aachen.de (M.B.); gunnar.seide@ita.rwth-aachen.de (G.S.); thomas.gries@ita.rwth-aachen.de (T.G.); 2 Institut für Kristallographie, RWTH Aachen (XTAL), Aachen 52056, Germany; E-Mail: roth@xtal.rwth-aachen.de

**Keywords:** poly(vinylidene fluoride), fiber, phase transition, bicomponent filament, poly(propylene), carbon nanotubes

## Abstract

This research explains the melt spinning of bicomponent fibers, consisting of a conductive polypropylene (PP) core and a piezoelectric sheath (polyvinylidene fluoride). Previously analyzed piezoelectric capabilities of polyvinylidene fluoride (PVDF) are to be exploited in sensor filaments. The PP compound contains a 10 wt % carbon nanotubes (CNTs) and 2 wt % sodium stearate (NaSt). The sodium stearate is added to lower the viscosity of the melt. The compound constitutes the fiber core that is conductive due to a percolation CNT network. The PVDF sheath’s piezoelectric effect is based on the formation of an all-trans conformation β phase, caused by draw-winding of the fibers. The core and sheath materials, as well as the bicomponent fibers, are characterized through different analytical methods. These include wide-angle X-ray diffraction (WAXD) to analyze crucial parameters for the development of a crystalline β phase. The distribution of CNTs in the polymer matrix, which affects the conductivity of the core, was investigated by transmission electron microscopy (TEM). Thermal characterization is carried out by conventional differential scanning calorimetry (DSC). Optical microscopy is used to determine the fibers’ diameter regularity (core and sheath). The materials’ viscosity is determined by rheometry. Eventually, an LCR tester is used to determine the core’s specific resistance.

## 1. Introduction

### 1.1. Poly(vinylidene fluoride) (PVDF)

Poly(vinylidene fluoride) (PVDF) is a fluoropolymer consisting of the monomer unit CF_2_–CH_2_. Furthermore, it is a polymorphic, semi-crystalline polymer showing at least four crystal phases at different processing conditions [1,2]. It has an excellent chemical stability and a large dipole moment of 9.8 × 10^−30^ cm, perpendicular to the polymer chain [3,4]. This results in piezo-, pyro-, and ferroelectric characteristics due to a polar crystal phase it can build. This phase is the β phase with an all-trans conformation in orthorhombic unit cells. It can be formed from the non-polar α phase. The most stable α phase forms from melt crystallization at temperatures below 160 °C and can be transformed into the β phase by application of mechanical stress at temperatures below 100 °C [2], as well as the application of high electric fields of about 18 MV/m [5]. Polarization in high electric fields will furthermore increase the piezoelectric effect when a β phase is readily available. In the β phase, the dipole moments in the unit cell are uniformly aligned due to the polarization process. This results in a net polarization, leading to a uniform alignment of the chains’ dipoles. The effect is a charge separation due to mechanical stress [1,6]. This polarization is a measure of degree of piezoelectricity. The polarization decreases again when heating the material.

PVDF films are already commercially available and are used as actuators and sensors in various cases [5,7,8].

### 1.2. Poly(propylene) (PP)

Poly(propylene) (PP) is a polyolefin that consists of the monomer unit propylene C_3_H_6_, widely used for melt spinning. It is capable of building different tacticities. The tacticity has an influence on the probability of crystallization and only the isotactic configuration with the highest likeliness to crystallize is suited for melt spinning [9].

### 1.3. Conductive Nanocomposites in Polymers

In general, there exist different approaches how to enhance polymer fibers in such a way that they are electrically conducting. The fibers can be coated with a metal (e.g., silver), to generate bleeder resistances of about less than 10^8 ^Ω. Another option is to use so called intrinsically conducting polymers (ICPs), which need to be doped with electron donors to be able to conduct electricity. Their disadvantage is that they have a low solubility and no meltability. Hence, they would have to be dispersed in a matrix polymer first [10]. The third approach is to use additives, which are incorporated into the polymer, such as conducting nanomaterial. In this case, it is realized by adding multi-walled carbon nanotubes (MWCNTs) to the polymer. MWCNTs consist of wrapped layers of graphene that consist of carbon atoms. The incorporation of MWCNTs into the polymer changes the polymer’s mechanical and electrical properties. In general, CNTs are very good conductors due to their graphite surface structure. Furthermore, the elastic modulus is in the region of 1 GPa [11]. Nano additives are used to systematically manipulate a material’s characteristics. Carbon Nanotubes (CNTs) in particular have an impact on a polymer’s conductivity [12]. CNTs are usually of the dimension of ~10–100 nm and the tube surface consists of pentagonally and hexagonally arranged carbon atoms [12,13,14]. Due to its graphite surface structure it has a very low resistivity. When a certain concentration within a polymer is reached it can generate elevated conductivity values. The transition for this effect happens when the percolation threshold is exceeded, above which the CNTs are close enough to each other to form conductive paths. For PP, this value is around 3 wt % [15]. The insertion of CNTs will eventually lead to an increased viscosity of a material [11,13,15].

However, for electrical applications, a polymer’s mechanical properties are to be conserved, and hence, one tries to add as low of an amount of CNTs as possible. Nevertheless, the CNTs have to form a network in order to be conductive within the polymer. Previously, different polymers with CNTs were used as core materials in bicomponent fibers. Cores consisting of PP as a base polymer yielded the best results. Impacts of CNTs on the behavior of polymers are various. CNT addition may result on better regulation of the melting process, *i.e.*, a sharp DSC peak in heat capacity at the melting temperature. The variable dimensions of CNTs have an impact on this behavior as well. In addition, the formation of phases in a polymer is influenced by CNTs. Furthermore, CNTs represent seed crystals resulting in a heterogeneous crystallization. All of these seeds are occupied first, before the normal crystallization process follows, shown by two peaks in a DSC cool-down process. As mentioned before, the addition of CNTs will result in an increased viscosity as well.

### 1.4. Melt Spinning and Bicomponent Fiber Extrusion

The production of synthetic fibers can be realized by thermoplastic extrusion, solution based extrusion, or electro spinning. Melt spinning is only possible for those polymers that do not degrade when melted. Melt spun PVDF leads to fibers that have characteristics in the same order of magnitude as other thermoplastic synthetic fibers, e.g., polyamide or polyester filaments. Among other things, these concern filament diameter, titre, or tensile strength. To minimize compatibility problems between two polymers, such as surface incompatibilities when extruded, it is important to adjust the viscosities to each other [9,16].

Conductive multicomponent fibers with piezoelectric capabilities were investigated, for example in [17], showing the spinnability of filler contents up to 10% (carbon black, multi-walled CNTs). Furthermore, the successful spinning of bicomponent fibers with piezoelectric properties has been achieved by different researchers. Lund and Hagström mainly concentrated on experiments incorporating carbon black into the PP core [18]. Lund *et al.* also manufactured bicomponent fibers with a polyethylene core and carbon black filling that gained peak-to-peak signal strengths of 40 mV under lateral compression [19].

Bicomponent fiber extrusion means the combination, and hence coextrusion, of two chemically and/or physically different polymers into one fiber. The arrangement of polymers can be of different geometry. In this case, as mentioned before, a spinneret is used that will produce a core/sheath bicomponent fiber. The spinneret’s cross section is designed in a way that both core’s and sheath’s center are at the same point, *i.e.*, the bores’ barriers are located on concentric circles [9].

In order to get the highest regularity for the fiber’s core and sheath diameter, the inner bore should end in the same plane as the spinneret’s surface, or it can even jut out slightly [9].

**Figure 1:**
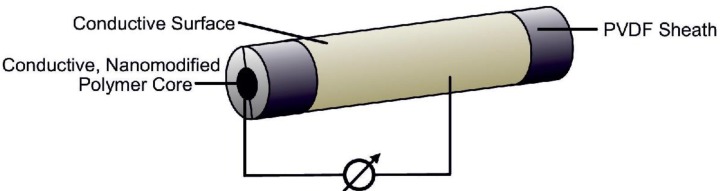
Schematic depiction of a melt spun bicomponent fiber with conductive poly(propylene) carbon nanotube (PP/CNT) core, poly(vinylidene fluoride) (PVDF) sheath and a conductive surface.

In analogy with single component spinning, the bicomponent fiber is led to a set of godets and to a winder. Differential rotation velocities of godet sets lead to the drawing of the filaments, resulting in the formation of a PVDF β phase. These fibers are eventually contacted (inner electrode: core, outer electrode: conductive cladding around sheath, *cf.* Figure 1 and used to measure electrical signals when deformed.

### 1.5. Sodium Stearate

Sodium stearate is a non-seizing compound with both hydrophilic and hydrophobic components. It decreases a plastic melt’s internal and external friction and, hence, viscosity. Its compatibility with a polymer depends on the polarity of both [20,21].

### 1.6. Aim of the Study

A combination of core and sheath material is sought, so that one yields a good piezoelectric capability in the sheath combined with a still satisfactory conductivity in the core. Eventually, the resulting fibers shall meet the requirements of a sensor fiber. The results shown will primarily explain the spinnability and the bicomponent fibers’ mechanical and physical properties, determined via different analytical methods. These methods include wide-angle X-ray diffraction (WAXD), differential scanning calorimetry (DSC), rheology, transmission electron microscopy, and optical bright field microscopy. In total, several combinations of core and sheath material were tested with respect to spinnability, yielding one combination giving the most promising results for further investigation. To make sure that the produced fibers can be used for sensor applications, these fibers will be tested for their piezoelectric and conductive properties.

## 2. Materials and Methods

The material defined first will undergo several processing stages until a synthetic bicomponent fiber is eventually formed. An overview of these steps and detailed information of the process parameters are provided.

### 2.1. Material

The material used as a sheath is the PVDF homopolymer SOLEF 1008 by Solvay Solexis. The main attributes of this polymer are a very low viscosity and a MFI of 0.8 g/min at 230 °C. In our study, this sheath is chosen to stabilize and strengthen the fiber because of a difficult to handle core component. The melting and crystallization point of the raw material is specified as 174 °C and 140 °C respectively [20].

The core base polymer is an isotactic polypropylene master batch PP2-T20 by Nanocyl, that already includes 20 wt % of NC7000 MWCNTs.

### 2.2. Compounding

To compound the core material, the twin-screw extruder Lab-Compounder KETSE 20/40 manufactured by Brabender GmbH & Co. KG, Duisburg is used. With its compact measure, it is a laboratory apparatus basing on two co-rotating screws with a maximum power of 11 kW and maximum revolutions per minute of 1200 rpm. The modular design of screw elements allows for matching different applications. The screws in use are the standard configuration by Brabender. After the compounding process, the strand is fed into a water quench for cooling. A granulator by Reduction Engineering GmbH ScheerPelletzing, Stuttgart eventually cuts the strand into pellets. The PP CNT master batch is died out with PP Moplen HP561, a pure polypropylene manufactured by LynodellBasell, until a 10 wt % CNT is reached. Parameters applied for compounding are temperatures of *T*_1_ = 160 °C and *T*_8_ = 190 °C. The temperature gradient of 30 °C is distributed over 8 independent heating zones, where heating zone 1 is where the granulate is inserted, and heating zone 8 is directly in front of the nozzle. The twin screws are set to rotate at 150 rpm. Furthermore, a 2 wt % sodium stearate is added.

### 2.3. Fiber Production

#### 2.3.1. Bicomponent Melt Spinning

**Figure 2:**
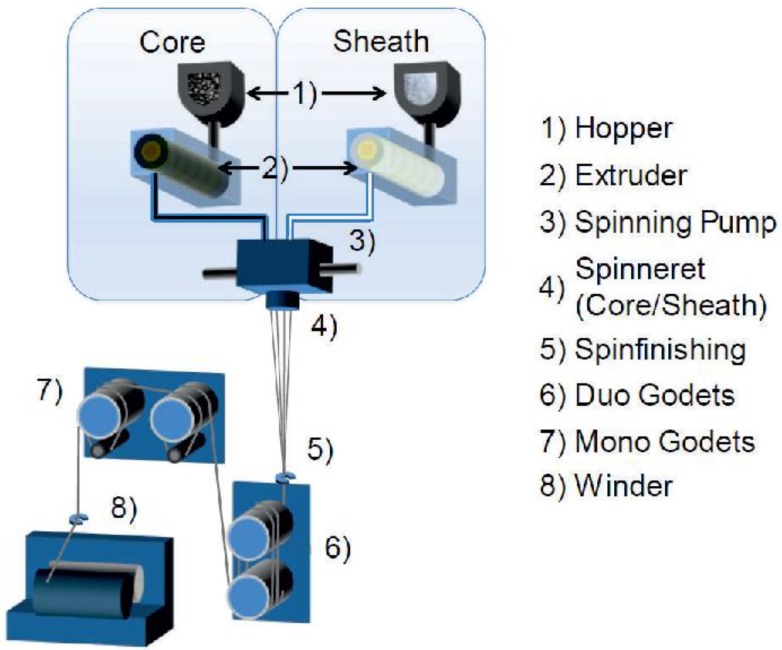
Bicomponent melt spinning plant. The Polymer granules are melted separately (1–3) and combined to a core/sheath structure in the spinneret (4).

The processing of thermoplastic polymers into fiber is commonly using melt spinning. In Figure 2, the path from granulate to a wound up fiber is shown. The PP used for the core is compounded with CNTs and sodium stearate before. The polymer granulate is being transported into an extruder for sheath and core respectively. The extruder temperatures can be set between 0 °C and 350 °C depending on the machine in use. The granulates are melted in the extruders. Each melt is transported through heated pipes, equipped with static mixers, to the spinning pumps. The pumps’ sizes are *V*_core_ = 0.3 cm^3^/revolution for the core material and *V*_sheath_ = 0.6 cm^3^/revolution for the sheath, respectively. Both pumps are set to the same speed of 10 rpm. Setting the pump speeds at a certain, well-defined rate, the extrusion velocities for each material “i” can be adjusted according to:

(1) $$v_{e,i}=\frac{Q_{\text{i}}}{\rho_{\text{i}}\cdot A_{\text{i}}}$$

depending on the mass-flow rates *Q*_i_, the materials’ densities *ρ*_i_ and the total capillary area *A*_i_. The polymers are pumped into the spin pack, where a metal screen filter is attached to for filtering reasons. Additionally, the filtering reduces the probability of having CNT agglomerations in the core material. After passing through the filtration stage the melts flow into the capillary of the monofilament spinneret, manufactured by Fourné Polymertechnik, Alfter-Impekoven. The capillary diameters are *d*_core_ = 0.2 mm and *d*_sheath_ = 0.6 mm, with a gap with diameters 0.2 mm (inner) and 0.35 mm resulting in capillary areas of *A*_core_ = 0.031 mm^2^ and *A*_sheath_ = 0.187 mm^2^. At the end of the capillary, the melt exits and forms a fiber. The temperature of the extrusion equipment, and thus the melt, is the major process parameter determining the viscosity and shearing of the polymers. Spinnability of the fibers for a core without NaSt was tested, but no stable spinning process was possible.

Eventually, the fibers are cooled down with a laminar air stream over a distance of 1500 mm, blowing perpendicularly to the fiber axis.

In Table 1, the parameters used for spinning the fibers are shown, including spinning pump settings, extrusion temperatures and laminar air settings. Eventually, a pair of duo godets takes up the melt. The adjustment of the godets causes the first stretching of the fibers. The ratio between the extrusion velocity for each core and sheath *v*_E,i_ and the winding velocity *v*_wind_ is defined as the melt draw ratio *MDR*_i_:

(2) $$MDR_{\text{i}}=\frac{v_{\text{W}}}{v_{\text{E},\text{i}}}$$

The winding velocity is set as low as possible to gain a best possible homogeneity for the core diameter. This is to result in a good conductivity.

**Table 1:** Process parameters for bicomponent melt spinning.

Spin Pump		Temperatures			Air Flow	
*N*_sheath_ [1/min]	*N*_core_ [1/min]	*T*_E,sheath_ [°C]	*T*_E,core_ [°C]	*T*_0_ [°C]	*T*_air_ [°C]	*V*_air_ [m/s]
10	10	260	240	245	23.0	0.50

The ratio between the extrusion velocities (extrusion velocity ratio, *EVR*) for sheath and core is independent of the winder velocity, and is defined as:

(3) $$EVR=\frac{v_{\text{E},\text{S}}}{v_{\text{E},\text{C}}}$$

All parameters for the melt drawing are shown in Table 2.

**Table 2:** Drawing parameters for bicomponent melt spinning.

Winder		Extrusion Velocities		*MDR*_core_	*MDR*_sheath_	*EVR*
*v*_winder_ [m/s]	*F*_fiber_ [cN]	*v*_E,sheath_ [m/s]	*v*_E,core_ [m/s]			
255.7	22.5	0.536	1.592	477.05	160.62	0.337

#### 2.3.2. Draw Winding

**Figure 3:**
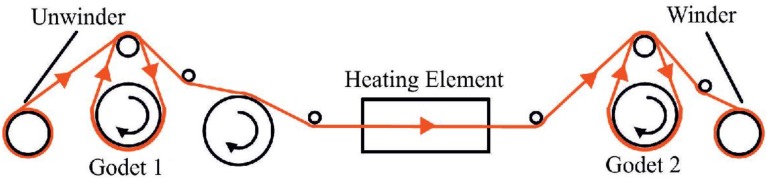
Drawing process as applied by DSM Xplore.

Draw winding is used for a continuous drawing of the fibers by godets, rotating at different velocities, which define the draw ratio. The draw unit Xplore is manufactured by DSM, Geleen, Netherlands. The machine consists of an unwinder, two godets, a heating unit in between these two godets, and a winder. The basic principle is shown in Figure 3. The drawing ratio is applied in between the two godets as the fiber is heated simultaneously. The temperature of the heating unit can be regulated between 0 °C and 300 °C. With the unit shown, a draw ratio of up to 10.0 can be realized.

For the experiments, a maximum draw ratio of 4.1 is possible. In total, five different draw ratios are applied additional to no draw ratio at all for comparison of the impact on the final fiber. At higher values than 4.1, the fiber would break. The temperature is kept constant at 140 °C, the speed of godet two is adjusted to constant 1000 cm/min. The draw winding parameters can be taken from Table 3.

**Table 3:** Parameters for applying different draw ratios.

Parameter	Value	[unit]
Godet 2 Velocity	1000	cm/min
Heating Unit Temperature	140	°C
Draw Ratio	1.00	–
	1.10	
	1.85	
	2.60	
	3.35	
	4.10	

A stable bicomponent fiber extrusion process could be realized. The produced fiber consists of a PVDF 1008 sheath and a compounded polypropylene core. The core contains 10 wt % of CNTs and 2 wt % of sodium stearate. The maximum draw ratio eventually applied without ripping fibers is 4.1. Limitations arise due to a constantly rising pressure inside of the spinneret. The time frame for continuous spinning is 30 min.

The optimal ratio between *A*_core_ and *A*_sheath_ is approximately 1:2. This is can be achieved by the appropriate choice of *N*_core_ and *N*_sheath_, according to *V*_core_ and *V*_sheath_. The extrusion velocities should be equal, but as a result of the above, the *EVR* is 0.337.

### 2.4. Characterization

The material’s viscosity is tested with a capillary rheometer of type Rheograph 25 by GöttfertWerkstoff-Prüfmaschinen GmbH, Buchen, Germany. The rheometry measurement shows if spinning can be easily carried out, since the spinnability depends on the viscosity at shear rates up to 10^5^ L/s.

All of the rheometry measurements are carried out at 250 °C. Two different capillaries are used, both with a diameter of 0.5 mm, but with different lengths, *l*_1_ = 5 mm and *l*_2_ = 20 mm.

Further thermal analysis is carried out using differential scanning calorimetry (DSC) for measuring heat flow differences during the process of heating, and subsequent cooling down. Due to this, conclusions can be drawn if a reaction is either endothermic or exothermic. In addition, DSC reveals information to identify the crystal phase involved in the observed transitions. The experiments were carried out with a Mettler Toledo DSC 1, equipped with a FRS5 sensor having 56 thermocouples. Conventional DSC was carried out with a heating rate of 2 K/min in the temperature range from −70 °C to 250 °C, leaving the sample for 5 min at each temperature.

Wide-Angle X-Ray Diffraction (WAXD) is used to examine the crystal structure and preferred crystallite orientation of the produced and processed fibers. The experiments are carried out with a single crystal diffractometer STOE IPDS II, equipped with an image plate for digital readout and two independent goniometer circles. The diffractometer uses a Mo-Kα X-ray tube, for which a voltage of 50 kV and a current of 25 mA are selected. The detector distance is chosen at 200 mm from the sample. The collimator width is 500 µm.

A special specimen holder for fibers allows reproducible results and the examination of the orientation distribution of crystals of the different phases. The exact position of the fiber sample in the X-ray beam is controlled by a CCD camera, positioned inside the diffractometer. The first goniometer circle, allowing rotations about the fibers axis, can be chosen arbitrarily because of the rotation symmetry of the sample. The second goniometer circle is used to tilt the fiber from a perpendicular incident of the radiation. A continuous rotation between 10° and 20° is chosen for recording the diffraction intensity of the (002)-plane in the α phase, and the (001)-plane in the β phase. These diffraction intensities are necessary to be determined in order to calculate the ratio between the α and β phase.

Intensity distribution from air scattering and signal noise are recorded in the same configuration, but with an empty specimen holder, and subtracted from the measured data. A software tool, developed at ITA, for processing the image plate data is capable of calculating intensity profiles in both directions 2θ and the azimuthal angle *φ* for arbitrary ranges in *φ* respectively 2θ with a definable integration range. Further evaluation of the integrated profiles and peak fitting is done with the Software OriginPro from Origin Lab Corporation, USA [22]. Uncertainties of the fitted values are calculated from the covariance matrix. Due to the fact that intensity values are determined by a counting process with a number of counts N, the errors of these values are calculated by determining $\sqrt{N}$.

Imaging techniques that are used are, on the one hand bright field microscopy, and on the other hand transmission electron microscopy (TEM). Optical bright field microscopy is carried out with the microscope DDM4000 M by Leica Microsystems CMS GmbH, Wetzlar, Germany. The software used to process the microscope data is calibrated in such a way, that it is true to scale. The magnification can be chosen between 12.5-fold and 500-fold. Hence, the data is analyzed to gain knowledge about the fiber diameter. For preparation, the fibers are cut carefully with a sharp razor in order not to deform it. Optical bright field microscopy is used to compare the values of calculated and measured core and sheath diameters to understand the impact of the draw ratio. A comparison of calculated and measured values is carried out.

The TEM measurements are carried out with the high-resolution apparatus Tecnai F20 by FEI, Hillsboro, USA. The magnification can be selected in a range between 100-fold and 500,000-fold. This is equivalent to resolving atomic scales of about 0.2 nm. In analogy with optical microscopy, in TEM one uses the electrons’ wave character. These electrons have wavelengths, five magnitudes less than visible photons. The samples have to be prepared in an ultra-microtome, Leica EM UC6. For TEM, the samples have to be cut in thin slices. The preparations for ultra-microtome cutting include embedding the fibers in epoxy, before eventually slices of about 100 nm are cut. Eventually, the impact of different draw ratios on the fibers’ specific resistivities is analyzed by using an LCR tester, model LCR-819 by RS Components Ltd., Corby, England. The maximum measurable resistivity is 100 MΩ. Measurements are carried out with alternating current at frequencies 0.1 kHz, 0.5 kHz, 1 kHz, 5 kHz, 10 kHz, 50 kHz, and 100 kHz. Five pieces of 50 mm length are cut from each fiber of different draw winding ratios, cutting the fibers slightly skewed in order to maximize the contactable core surface. A piece of removable tape is wrapped around the centre, leaving the edge blank. The fibers are sputtered with gold and the tape is subsequently removed again. The fibers are contacted in the LCR tester and the alternating current was applied. The fiber cores’ specific resistivities were calculated via:

(4) $$\rho =R\cdot \frac{\pi \cdot D_{\text{core}}^{2}}{4\cdot L_{\text{fiber}}}$$

## 3. Results and Discussion

### 3.1. Rheometry

Rheometry measurements have excluded certain polymers and their mixtures in preceding tests due to some of the materials tested hardening within the rheometer’s nozzle.

**Figure 4:**
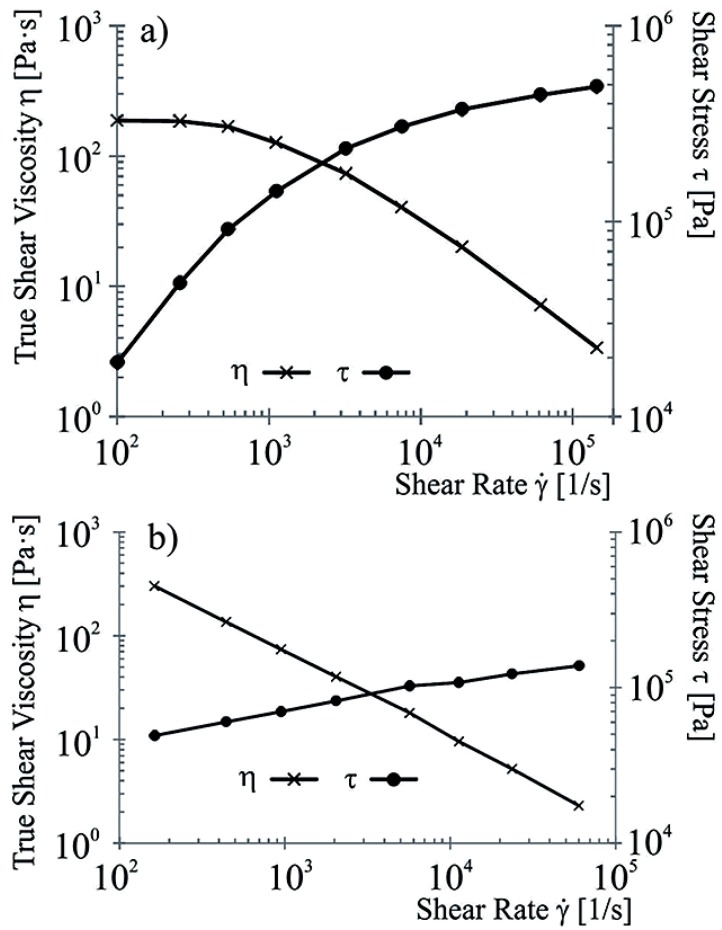
Shearing behavior for both the sheath material PVDF 1008 (**a**) and the core material PP/CNT/NaSt (**b**).

Each of the shear rates given for sheath and core are determined due to the necessary process parameters for the material tested, and hence the viscosity is measured. Figure 4a shows the results for the sheath material PVDF 1008. The viscosity decreases disproportionately as the shear rate increases. The materials behavior is shear thinning. At shear rates relevant for melt spinning up to 10,000 L/s, the viscosity is low enough (*η* = 10 Pas). Figure 4b shows the behavior of the core material. The modification with CNTs results in the disappearing of shear thinning, as would be expected for unmodified polypropylene. In the case of CNT modified PP, the viscosity drops linearly with increasing shear rates. Both measurements are carried out at 250 °C. A comparison between the 10 wt % CNT plus 2 wt % sodium stearate PP and the standard PP without any modification shows, that equal viscosities are reached for shear rates of 48,808 L/s for the former, and 50,930 L/s for the latter, that is only a 4% drop in applicable shear rates resulting in the same viscosity.

### 3.2. Differential Scanning Calorimetry (DSC)

**Figure 5:**
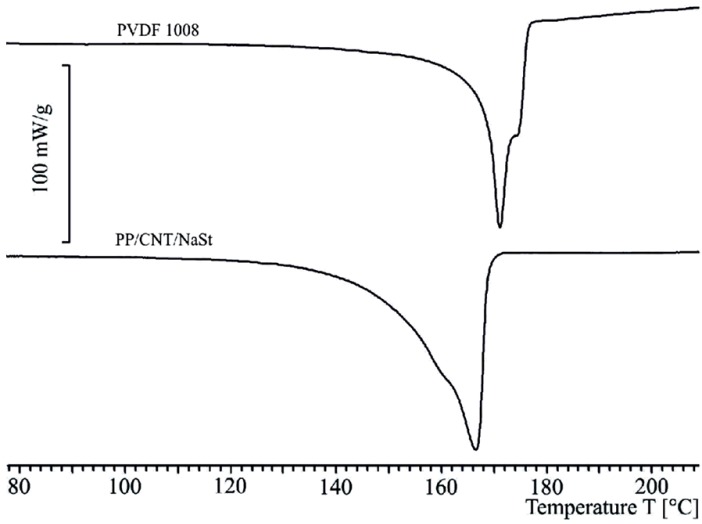
Differential scanning calorimetry (DSC) results, independently measured for the sheath material and core compound.

In Figure 5, the results of DSC analyses for the sheath and the core compound are depicted. Both components are necessarily required for the spinning of the resulting conducting bicomponent fiber. An analysis of the diagram provides that PVDF 1008 melts in a range of temperature of *ca.* 155 °C to 175 °C and the core melts in a region of *ca.* 140 °C to 170 °C. Due to overlapping regions in which both polymers melt, there is only a limited possibility to evaluate the results concerning fusion enthalpy of fibers made of these polymers. Hence, crystallinity is determined via X-ray diffraction.

**Figure 6:**
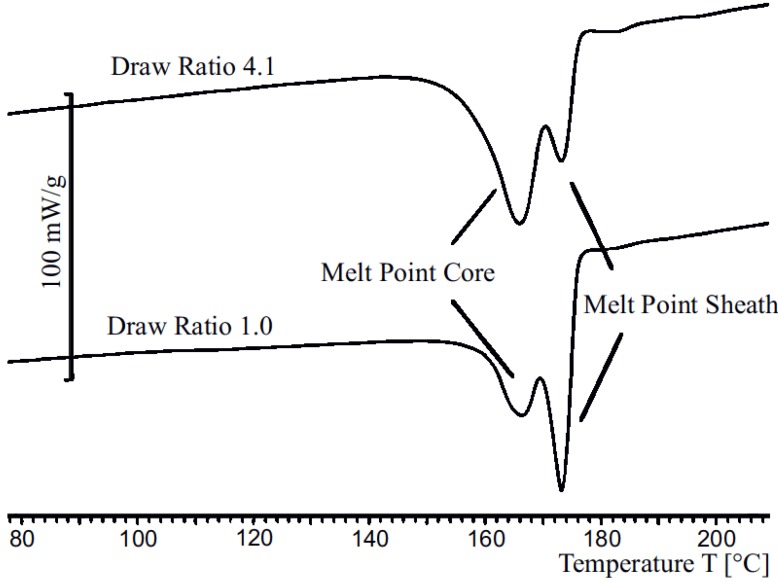
DSC results for undrawn and drawn fibers at draw ratios 1.0 and 4.1 respectively.

In Figure 6, the differential scanning calorimetry measurements of bicomponent fibers with draw ratios of 1.0 and 4.1 are presented. Apparently, for a draw ratio of 1.0 there exists a baseline shift. This is caused by the low heat conductivity of PVDF (0.2 W/m·K). Due to this effect, the PVDF sheath has an increased insulating effect, and therefore there is a time delay during the melting process for the core. Additionally, the polymer melts in a broader temperature region. These effects cause the aforementioned baseline shift.

The fiber with a draw ratio of 4.1 has a significantly thinner sheath. Due to the reduction of the thickness of the sheath, the baseline aligns with the original one and the insulation effect decreases. However, due to this shift, quantitative analyses are almost impossible. The problematic nature of the similar melting temperatures—as it can be expected by evaluating Figure 6—can be obtained easily by taking a look at the DSC diagram of both materials. In the diagram both areas of the materials for sheath and core are overlapping and so formerly distinct peaks cannot be separated from each other and, therefore, even qualitative conclusions cannot be drawn.

Nevertheless, evaluating the height of the core-peak allows to state that the melting enthalpy of the core’s material combination is increasing. This effect can be explained in such a way that there is only less orientation in crystal phase of the core after the spinning process. Increasing the stretching factor, the amount of orientation appreciates in value and therefore the peak is higher.

### 3.3. Wide-Angle X-Ray Diffraction (WAXD)

**Figure 7:**
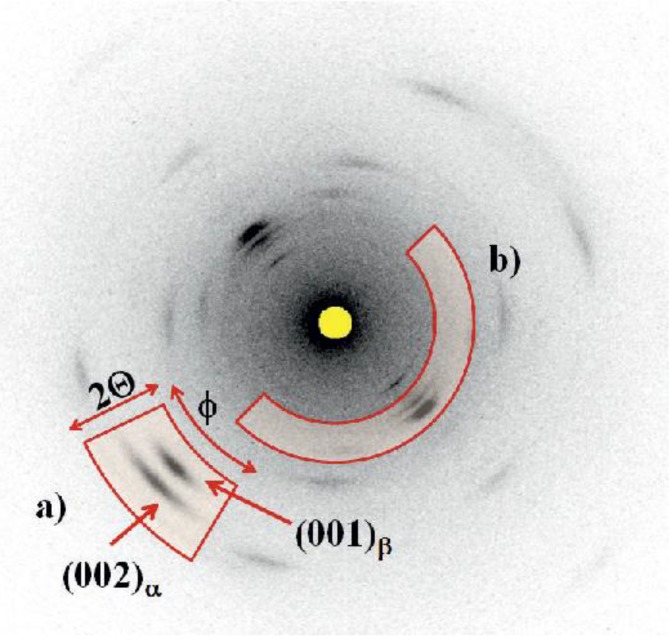
Wide-Angle X-Ray Diffraction (WAXD) regions of interest that are analyzed for determination of β ratio (**a**), and orientation factor of crystalline and amorphous regions (**b**).

Intensity profiles from region (a) in Figure 7 (draw ratio 2.6) contain the intensity peaks of the (002)-lattice planes from the α phase and the (001)-peak from the β phase. A draw ratio of 2.6 is chosen exemplary because, in this case, almost equal mass fractions of α and β phase are available. For each crystalline peak, a Gaussian peak, with the intensity distribution:

(4) $$I(x)=\;\frac{1}{\sqrt{2\pi }\cdot \sigma }\exp(-\frac{1}{2}\frac{(x-\mu )?}{\sigma^{2}})$$

is fitted, where *μ* is the mean and *σ* is the standard deviation.

The intensity areas of the peaks are used to calculate the mass ratio of β phase in the sample. It might be calculated via:

(5) $$I_{\text{rel};\beta }\;=\;\frac{I_{\beta }}{I_{\alpha }+I_{\beta }}$$

**Figure 8:**
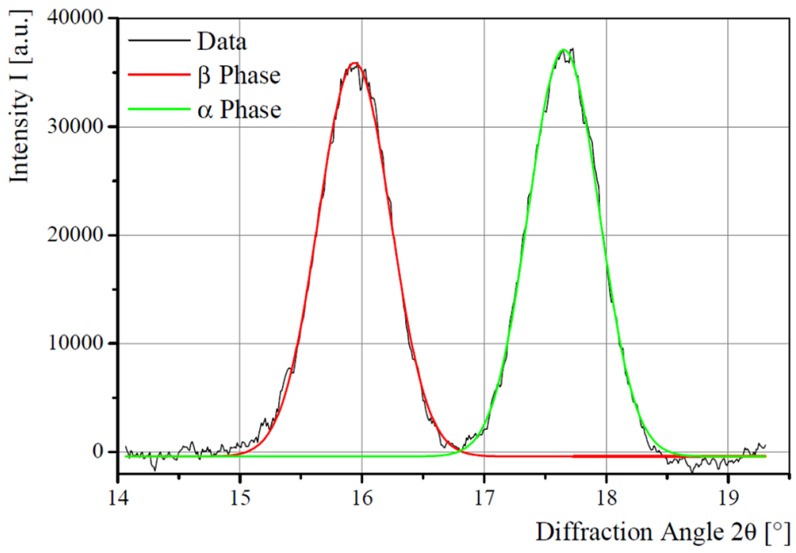
Gauss fits to bicomponent fiber WAXD data. The analyzed intensity distributions are for the (001)β and (002)α.

**Figure 9:**
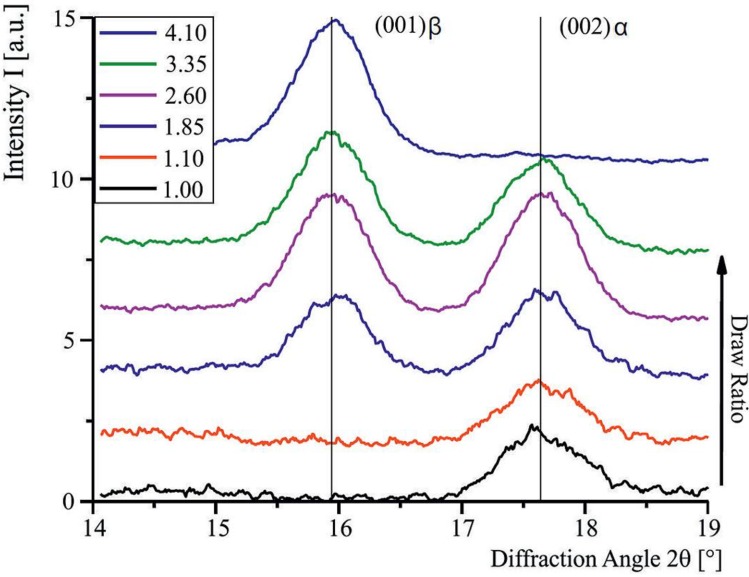
α and β phase intensity distributions for fibers drawn at different draw ratios.

**Figure 10:**
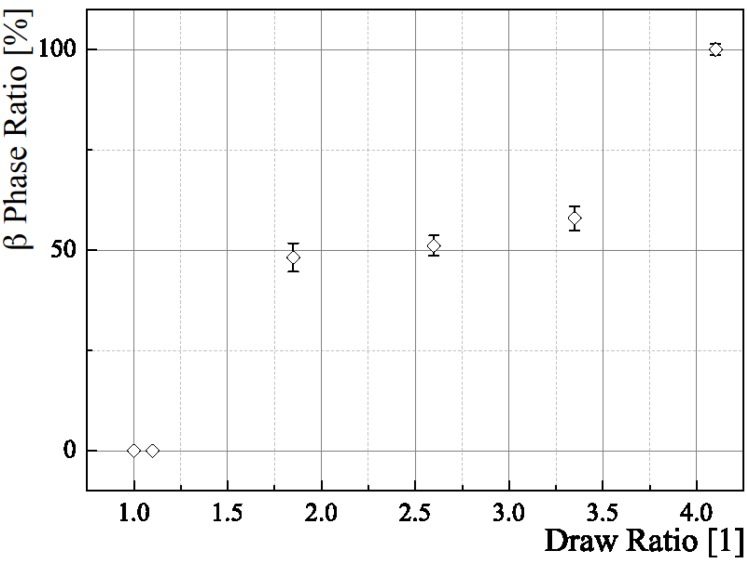
Sheath β phase content at different draw ratios.

Figure 8 shows the (002)α/(001)β data for a draw ratio of 2.6, resulting in *I*_β_ = 51.08% ± 0.52%. The according plots for all draw ratios are depicted in Figure 9. Figure 10 shows the different β phase mass fractions plotted against the according draw ratios, underlining the fact, that an increasing draw ratio and, hence, increasing mechanical stress at temperatures around 140 °C leads to the formation of β phase. The result of this analysis is that a maximum draw ratio of 4.1 is crucial for the production of sensor fibers to have a high enough β-mass fraction. At this maximum draw ratio, the α phase is completely transformed into β phase. The results are in accordance with previous research [5,19,20].

For the orientation distribution deduced from region (b) in Figure 7, a Gaussian intensity profile is assumed for the crystalline phases having well resolved peaks. For the non-crystalline regions, a second Gaussian profile, and a constant intensity offset, are chosen.

The total area of the profile and the offset are assumed to be caused by the non-crystalline regions and must be considered when calculating the orientation factor. The calculated profiles *I*_calc_ are used for the calculation of the standard deviation of the azimuthal angle <sin^2^*φ*>:

(6) $$<{\text{sin}}^{2}\varphi >=\frac{\int_{0}^{2\pi }{\text{sin}}^{2}\varphi \;I_{\text{calc}}(\varphi )d\varphi }{\int_{0}^{2\pi }I_{\text{calc}}(\varphi )d\varphi }$$

The obtained values can be used to calculate the orientation factor for crystalline and non-crystalline phases [22]:

(7) $$f=1-\frac{3}{2}<{\text{sin}}^{2}\varphi >$$

An orientation factor equal to 1 would mean a perfect alignment of the polymer chain in the same direction. The increasing draw ratio also leads to an increased orientation factor for the crystalline regions of the fiber. Values are given in Table 4. For draw ratios 1.00 and 1.10, no β phase has formed yet, hence it’s orientation factor cannot be determined, as is the case for a draw ratio of 4.1 with respect to the α phase.

**Table 4:** Values for orientation factors of α and β phase. Standard deviations *σ*_f,i_ are given.

DR	*f*_α_	*σ*_f,α_	*f*_β_	*σ*_f,β_
1.00	0.6805	0.0130	–	–
1.10	0.6644	0.0153	–	–
1.85	0.9558	0.0045	0.9911	0.0004
2.60	0.9831	0.0008	0.9921	0.0002
3.35	0.9828	0.0008	0.9904	0.0003
4.10	–	–	0.9937	0.0002

### 3.4. Bright Field Microscopy

**Figure 11:**
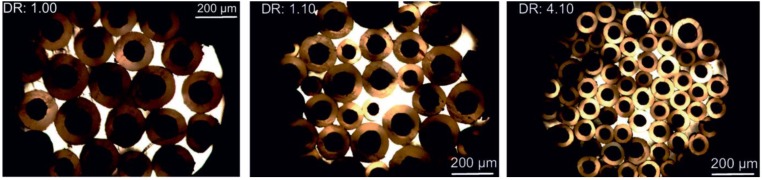
Optical bright field microscopy images for different draw ratios. The fibers’ center is dark, the PVDF sheath is brighter.

Figure 11 shows optical bright field images for different draw ratios. With a 75-fold magnification, one can see the different distribution of diameters and circular fiber shapes. The image indicates the occurring irregularities concerning uniformity for the bicomponent fibers, with an increasing regularity for larger draw ratios.

For core and sheath each, the calculation of theoretical fiber diameters is done by calculating the mass per unit length first via:

(8) $$m_{\text{L}}=\;\frac{Q}{v_{\text{w}}}$$

where *v*_w_ is the speed of the winder and* Q* is the mass flow rate. Hence, the area for core and sheath at given material density *ρ* each is calculated by

(9) $$A=\frac{m_{\text{L}}}{\rho }$$

**Figure 12:**
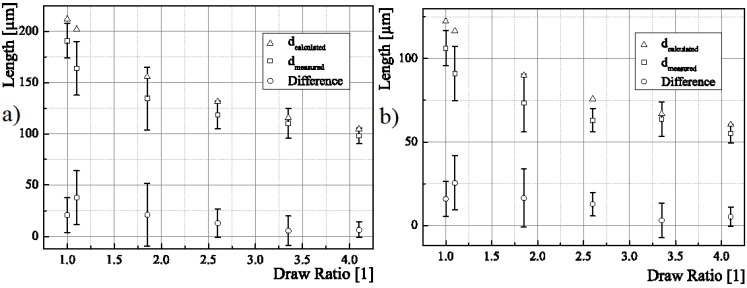
Measured diameters from bright field imaging for (**a**) core only and (**b**) the whole fiber, each at different draw ratios.

Hence, core and fiber diameters *d*_c_ and *d*_f_ can be calculated via

(10) $$d_{c}=2\cdot \sqrt{A_{c}/\pi }$$

(11) $$d_{\text{f}}=2\cdot \sqrt{(A_{c}+A_{s})/\pi }$$

with values *A*_c_ and *A*_s_ as calculated in Section 2.3.1. Table 5 shows the calculated and the measured values for sheath and core diameters. The values underline the first impression of a highest regularity of sheath and core diameters for increasing draw ratios, as the standard deviations are lowest for a draw ratio of 4.1. Interesting to see is that the standard deviations have a peak for a draw ratio of 1.85, decreasing again for higher draw ratios. This result can be explained by the occurrence of stress peaks in the already irregular parental fiber, resulting in a lengthening of thin regions due to a higher probability of the orientation of polymer chains therein. Another explanation is the raising of CNT agglomerations. Depending on a fluctuation of necessary forces to raise agglomerations, different draw ratios will lead to different forces. Hence, the CNT agglomerations are probably raised over the whole draw ratio range. For higher draw ratios, the standard deviations decrease for both sheath and core diameter, resulting in a more regular fiber. Furthermore, the diameters themselves decrease due to the drawing of the fibers. Though, the calculated values for the diameters show a systematic deviation towards higher values than those measured. In Figure 12, the diameters are shown *versus* the draw ratios applied to the fibers each for the core and the whole fiber. The plot shows a decreasing slope for increasing draw ratios.

**Table 5:** Calculated and measured values for sheath and core diameters as well as statistically determined standard deviations.

CDR	Calculated	Measured			Calculated	Measured		
	*d*_f_	*d*_f_ [µm]	*σ*_d,f_ [µm]	%	*d*_c_	*d*_c_ [µm]	*σ*_d,c_ [µm]	%
1.00	211.7	190.9	17	8.9	122.2	106.2	10.5	9.9
1.10	201.8	163.9	26.2	16.0	116.5	91	16.3	17.9
1.85	155.6	134.5	30.6	22.8	89.9	73.4	17.4	23.7
2.60	131.3	118.5	13.6	11.5	75.8	63	7.1	11.3
3.35	115.7	110.2	14.4	13.1	66.8	63.7	10.2	16.0
4.10	104.5	98.2	7.6	7.7	60.4	55.1	5.6	10.2

### 3.5. Transmission Electron Microscopy

**Figure 13:**
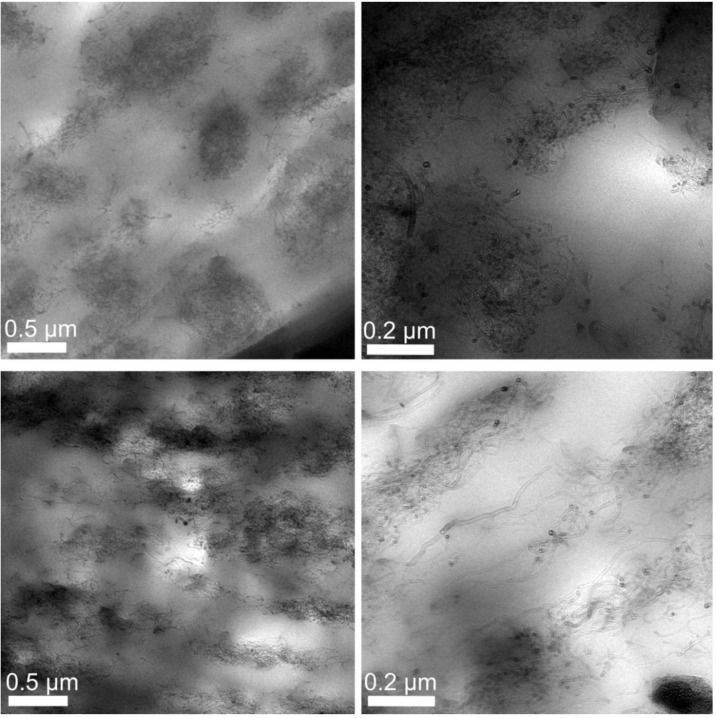
TEM images showing the CNT percolation network at different resolutions and draw ratios. Top: No Drawing applied. Bottom: Draw ratio 4.10.

Figure 13 shows the comparison of TEM pictures for the draw ratios 1.00 and 4.10. For the former fiber, one can see that no regular distribution of the CNT network is the case. The top right image shows at a higher resolution that no preferred orientation of the single CNTs occurs either.

A reason for the irregular CNT distribution might be due to the compound that consists of the 20 wt % CNT and PP master batch and the pure PP, resulting in insufficient mixing. On the other hand, domains are built that might result in conductive paths. The images at the bottom show the influence of the draw winding process. An orientation of the CNT network following a path from left to right of the images is visible. However, the CNTs are constrained to domains of higher concentration, nevertheless these domains are stretched. The bottom right image shows single CNTs at a higher resolution, where the CNTs’ orientation from image left to image right is visible. Furthermore, the gaps between the domains are populated with CNTs, which seem ripped out of the agglomerations. Comparing the TEM images at different resolutions for two different draw ratios, the conclusion to be drawn is that an increased draw ratio has a large impact on the orientation of the CNTs, as well as the shape of the CNT domains. This process leads to the assumption of an improved conductivity, yet to be tested.

### 3.6. Specific Resistivity

**Figure 14:**
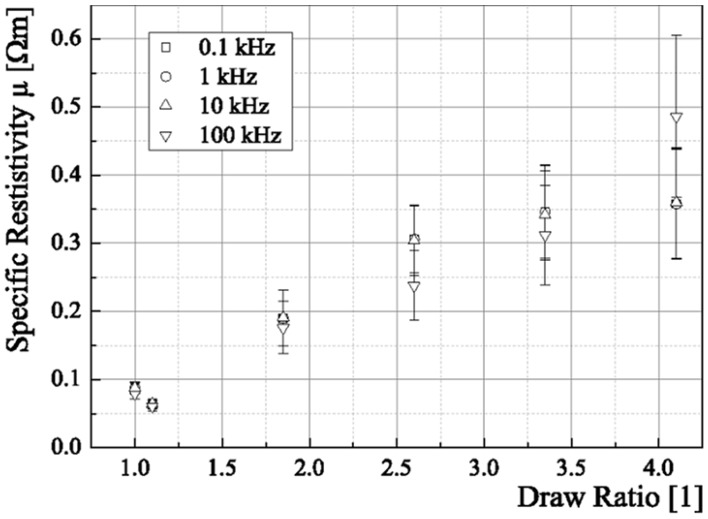
Behavior of fiber resistivity with increasing draw ratio for different applied frequencies.

The results for testing the different specific resistivity of fibers different draw ratios were applied to are shown in Figure 14. Obviously, with increasing draw ratio the specific resistivity increases. This is the case except for the draw ratio 1.1. Explanations for both are on the one hand, that an increasing draw ratio will lead to a decreased core diameter, minimizing the effective conductive cross section. The drop in specific resistivity for a draw ratio of 1.1 can be explained by the fact that the diameter has not significantly been decreased yet, but the processing itself (tempering, *i.e.*, applying a temperature of about 140 °C) when drawing the fiber might have a positive influence on the fiber’s conductivity, according to the results in [18]. Tempering is not applied to the undrawn fiber, and hence this step is the only other different treatment that could explain the discrepancy between the fiber without drawing and the fiber with a draw ratio of 1.1. The impact of frequency variation is that an increasing frequency for *f* > 50 kHz will result in lower resistivities. Except for the fiber with draw ratio 4.1, where the resistivity increases above 50 kHz. Nevertheless, the standard deviations allow for the fibers at different frequencies but same draw ratios an agreement within 1-σ.

## 4. Conclusions and Outlook

It can be stated that the used material combination is suitable for the manufacturing of bicomponent fibers. The used materials were PVDF 1008 for the sheath and a polypropylene by Nanocyl including 20 wt % of NC7000 MWCNTs that was died out with Moplen HP561 for the core, added by 2 wt % sodium stearate as a non-seizing compound. A stable spinning process is possible within 30 minutes of no interruption, constrained by the need of changing bobbins. Furthermore, the draw winding process for establishing the β phase in the PVDF sheath was successful resulting in 100% β-ratio at a draw ratio of 4.1.

Nevertheless process and material improvements need to be realized. For example, adjusting the matrix viscosity by further investigating the impact of a non-seizing compound still offers opportunities. In addition, the influence of high shear rates around 63,000 L/s on the CNT distribution influence is yet to be determined. Basing on the results, a new spinneret can be designed, including the desired flow rate. Adjusted viscosities and extrusion velocities for each sheath and core material need to be considered. A new conception of the filter can lead to a prolonged stability of the spinning process. At this point, it has to be taken into account that the CNT agglomerations will not clog the nozzle.

A winder with reduced velocity can be used to gain larger fiber diameters for an improved conductivity, building a better fundament before due to draw winding the diameter is decreased again. Depending on the decreased resistivity for a draw ratio of 1.1, it seems necessary to further analyze the tempering process and its impact on the cores’ resistivity.
